# Exploring the Role of Interleukin-6 Receptor Inhibitor Tocilizumab in Patients with Active Rheumatoid Arthritis and Periodontal Disease

**DOI:** 10.3390/jcm10040878

**Published:** 2021-02-20

**Authors:** Codrina Ancuța, Rodica Chirieac, Eugen Ancuța, Oana Țănculescu, Sorina Mihaela Solomon, Ana Maria Fătu, Adrian Doloca, Cristina Iordache

**Affiliations:** 1 Department of Rheumatology, Faculty of Medicine, Grigore T. Popa University of Medicine and Pharmacy, 700115 Iasi, Romania; codrina_ancuta@yahoo.com; 2 Sanocare Medical and Research Center, 700503 Iasi, Romania; chiriac01ro@yahoo.com; 3 Research Department, Elena Doamna Clinical Hospital, 700398 Iasi, Romania; eugen01ro@yahoo.com; 4 Department of Odontology-Periodontology, Fixed Prosthodontics, Faculty of Dental Medicine, Grigore T. Popa University of Medicine and Pharmacy, 700115 Iasi, Romania; 5 Implantology, Removable Dentures, Dental Technology Department, Faculty of Dentistry, Grigore T. Popa University of Medicine and Pharmacy, 700115 Iasi, Romania; anafatu2007@yahoo.com (A.M.F.); ccmiiordache@yahoo.com (C.I.); 6 Department of Preventive Medicine and Interdisciplinarity, Faculty of Medicine, Grigore T. Popa University of Medicine and Pharmacy, 700115 Iasi, Romania; adrian.doloca@umfiasi.ro

**Keywords:** rheumatoid arthritis, periodontal disease, tocilizumab

## Abstract

Background: The aim of our study was to explore the influence of weekly subcutaneous administration of interleukin-6 (IL-6) receptor inhibitor tocilizumab (TCZ) on periodontal status in a local longitudinal study of patients with rheumatoid arthritis (RA) and periodontal disease (PD). Methods: We performed a 6-month prospective study in 51 patients with chronic periodontitis and moderate-to-severe RA starting TCZ in accordance with local recommendations. Extensive rheumatologic (clinical activity, inflammatory, serological biomarkers) and periodontal (visible plaque index, gingival index, bleeding on probing, probing pocket depth, clinical attachment loss) assessments were done. Changes in RA activity and periodontal status were reassessed after 3 and 6 months. Results: We demonstrated significant correlations between periodontal status, disease activity, and serologic biomarkers (*p* < 0.05). Tocilizumab significantly improved the gingival index scores and decreased the number of sites with bleeding on probing after only 3 months (*p* < 0.05), while the probing pocket depth significantly decreased after 6 months; overall, clinical attachment loss presented only slight changes without any statistical significance as well as teeth count and plaque levels (*p* > 0.05). Conclusion: IL-6 inhibition is able to improve periodontal outcomes in patients with RA and concomitant PD, which is essentially related to a dramatic decrease in serum inflammatory mediators.

## 1. Introduction

### 1.1. Rheumatoid Arthritis and Periodontal Disease Association

Rheumatoid arthritis (RA) is an autoimmune condition characterized by joint inflammation and destruction associated with chronic systemic inflammation accounting for significantly impaired quality of life. It is defined by the excessive activation of proinflammatory cytokines mediators, specific autoantibodies and progressive irreversible articular damage [1,2]. Considered the hallmark of RA and, perhaps, the most important step in the pathobiology of the disease, the immune response against citrullinated peptides is driven, at least in part, by antigens derived from the periodontal tissue exposed to a dysbiotic oral microbiome during periodontitis [1,2,3,4].

Chronic periodontitis is a complex condition outlined by chronic inflammation and the subsequent damage of soft collagen-rich tissues progressing to periodontal ligament and alveolar bone loss as well as a gradual increase in tooth mobility [1,2,3,4,5,6,7,8,9]. It is typically initiated by an infection with oral anaerobic bacteria followed by inflammatory and immune response in the gingival and periodontal microenvironment [1,2,3]. Autoantigens generated during citrullination induced by peptidylarginine-deaminase enzyme produced by *Porphyromonas gingivalis (P. gingivalis),* the keystone pathogen in the oral microbial biofilm, break the immune tolerance with induction of anti-citrullinated protein antibodies (ACPA) and promote chronic inflammatory response in both periodontal and synovial/articular tissues [1,3,4,10,11,12,13,14,15,16,17,18].

Periodontal disease (PD) is generally associated with a broad spectrum of chronic systemic disorders including diabetes, cardiovascular, respiratory, kidney, and neurodegenerative diseases as well as immune-mediated rheumatic conditions [1,2,3,4,5,19]. Several epidemiological studies have already communicated that PD is more prevalent during RA and vice versa [4,20,21]. Indeed, patients with PD have an increased risk to develop RA, compared to general population, particularly those with a long history of more severe periodontitis, mostly explained by excessive protein citrullination [14,21]. Furthermore, it seems that *P. gingivalis* positive-periodontitis is more likely to occur in ACPA-positive individuals without any arthritis, suggesting that PD may precede RA [4,11,15,20,22]. On the other hand, RA patients experience a greater risk of PD, irrespective of disease duration, especially in ACPA-positive subtype [1,2,3,6,7,8,11,14,19]; moreover, they are prone to develop moderate to severe periodontitis in established compared to early disease [6,7,8,9,14,19,23]. A detailed analysis of periodontal status in first-degree relatives of RA cases discovered a higher prevalence and severity of periodontitis in ACPA-positive RA [9,14,22,23]. Altered periodontal condition during RA seems to be multifactorial, related to increased serum concentrations of proinflammatory cytokines and altered motor skills of the rheumatoid hand which can also contribute to compromised oral hygiene [1,4,5,22].

This intriguing relationship between PD and RA is roughly supported by similar pathogenic pathways in a genetically predisposed host (human leukocyte antigen HLA-haplotype DRB1, HLA-DRB1, shared epitope) triggered by common environmental risk factors (cigarette smoking) [1,2,4,10,18,24,25,26]. Important pathobiologic processes refer to the overexpression of proinflammatory cytokines (tumor necrosis factor alpha—TNF-α, IL-1β, IL-6 and IL-17), inflammatory mediators (prostaglandin E2, nitric oxide) and degradation enzymes (matrix metalloproteinases 1, 8, 9, and 13), osteoclast activation, and progressive articular and alveolar bone damage [1,2,4,10,18,23,24,25,26]. Considered as the cytokine signature, the aberrant activation of TNF-α and IL-6 regulates immune response and bone metabolism in RA [1,3,5,6,16]; high concentrations of both cytokines were detected in serum, synovial tissues, as well as synovial fluids [18,27], positively correlating with disease activity [28]. Different studies have also confirmed higher levels of potent IL-6 and TNF-α in inflamed gingival tissues, gingival crevicular fluid, and serum in patients with PD than in the healthy controls [17,28,29,30,31,32,33]. Moreover, increased TNF concentrations are associated with less favorable periodontal indices such as bleeding on probing (BOP), probing pocket depth (PPD), and clinical attachment loss (CAL), while serum IL-6 concentrations decreased following periodontal treatment [17,27,28,32,34]. Surprisingly, salivary levels of TNF-α, IL-6, IL-8, and IL-17A can be affected not only by periodontitis but also in RA [35,36]. Furthermore, the interplay between the subgingival biofilm, particularly PD-associated pathogens, and the host immune system may contribute to both PD and RA [1,2,18].

### 1.2. The Role of Different Therapies on Rheumatoid Arthritis and Periodontal Disease Outcomes

The evolving model for dynamic interrelation between RA and PD encourages the concept that standard management for RA may be effective in improving the outcomes in PD and vice versa [4,7,14,19,37,38,39]. Pivotal studies have already explored the role of different synthetic and biological therapies in active RA and comorbid periodontal disease, showing controversial results [5,9,12,16,17,25,28,35,36,37,38,39]. Overall, there is a trend to consider that TNF inhibitors, IL-6 receptor antagonist, B-cells depletive agents and, even, JAK inhibitors improve periodontal health in both RA and other arthritis (e.g., ankylosing spondylitis, psoriatic arthritis); it seems that all these drugs are ultimately effective in decreasing gingival and periodontal inflammation and, to a lesser extent, associated tissue damage [16,27,28,35,36,37,39,40,41,42,43,44,45,46,47,48,49,50]. Researchers even proposed a multistep approach of the sequential tissue repairing following TNF inhibitors, comprising reduced leukocytes traffic in the inflamed tissue, decreased proteolytic activity, and the normalization of osteoclast activity [1,5,9,12]. However, there are differences among anti-TNF agents as only adalimumab and etanercept significantly improved periodontal outcomes in as rapid as six months, while infliximab worsened gingival inflammation but prevented gingival bone loss [5,14]. Furthermore, according to a study by Kobayashi and colleagues, tocilizumab (TCZ) also ameliorates periodontal inflammation in RA with periodontitis as TNF inhibitors do [16,17,27,28,31,32,36,37]. Its beneficial effects were potentially explained by the decrease in TNF-α serum levels as well as immunoglobulin G and serum amyloid along with a consistent impact on serum inflammatory mediators and indirect influence on periodontal inflammation [16,17,27,28,40].

On the other hand, several papers have addressed the effect of specific periodontal therapies (e.g., non-surgical scaling and root planning) on clinical RA activity in patients with chronic periodontitis with controversial results [37,51,52,53,54,55,56,57]. The most recent data from the ESPERA (Experimental Study of Periodontitis and Rheumatoid Arthritis) cohort failed to demonstrate clinical improvement in established RA following aggressive and intensive periodontal treatment [57].

Considering the gap in the literature regarding the role of anti-rheumatic drugs on periodontal outcomes, the aim of our study is to assess the influence on the periodontal status of weekly subcutaneous administration of tocilizumab in a local group of patients with rheumatoid arthritis and chronic periodontitis.

## 2. Materials and Methods

### 2.1. Study Design and Population

We performed a prospective longitudinal study in fifty-one patients with moderate-to-severe RA and insufficient response to either conventional synthetic or biologic disease-modifying antirheumatic drugs (DMARDs), starting TCZ according to the local recommendation for biologic and targeted synthetic therapy aligned with European League Against Rheumatism (EULAR) consensus statement and guidelines.

We performed extensive rheumatologic and full mouth assessments at baseline (before the first administration of TCZ) as well as after 3 and 6 months of therapy.

### 2.2. Inclusion Criteria

The patients were aged 18 and older, able to give informed consent themselves and to participate in the study, and willing to forgo any optional examinations.

Patients fulfilled either the old 1987 American College of Rheumatology (ACR) criteria or the new 2010 classification criteria of ACR and EULAR and were followed up in one academic rheumatology department in Northeast Romania over a period of 3 years (July 2017–January 2020).

### 2.3. Exclusion Criteria

Several exclusion criteria were applied before enrollment in this study because of their potential interference with a correct evaluation of periodontal status, as follows: ex- or current smokers, pregnant and breastfeeding women, patients with diabetes mellitus, implants, poorly fitting fixed and/or removable prosthodontics and fewer than eight evaluable teeth, patients receiving systemic or local antimicrobials, antiplatelet drugs, any type of anti-inflammatory medication or periodontal therapy within the previous 3 months.

A total of sixty-eight patients were eligible for and received TCZ for their active RA; however, among them, seventeen had no oral issues at baseline evaluation and were excluded from the study.

### 2.4. Ethical Considerations

The baseline clinical documentation of periodontitis cases was collected in the context of routine check-up in the dental clinic of Sanocare Medical and Research Center.

The study was approved by the local ethics committee (Sanocare Medical and Research Center, Prot. No 15/12.12.2016) and was found to conform to the guidelines of the Declaration of Helsinki. Written informed consent regarding the use of the collected data in the context of training and research was signed by all the participants before enrollment. The data used in the study were anonymized. According to the U.S. Department of Health and Human Services (HHS) definition, this investigation is not considered human subjects research.

### 2.5. Rheumatologic Assessments

RA-related variables comprised clinical (tender and swollen joint count based on a 28-joint assessment, 0–10 cm visual analogue scale, VAS, pain), inflammatory tests (erythrocyte sedimentation rate, ESR, and C-reactive protein, CRP) as well as disease activity scores calculated on DAS28-CRP (Disease Activity Score on 28 joints using C-reactive protein) and SDAI (Simplified Disease Activity Index) were performed at all three visits.

DAS28-CRP was calculated with a formula that considered the tender and swollen joints, the patient’s general assessment of their condition scored on a visual analogue scale (VAS), and CRP. DAS28-CRP comprises four categories: remission (DAS28-CRP < 2.3), low (2.3 ≤ DAS28-CRP < 2.7), moderate (2.7 ≤ DAS28-CRP < 4.1), and high disease activity (4.1 ≤ DAS28-CRP).

Designed as the numerical sum of five outcome parameters (tender and swollen joints, patient, and physician global assessment of disease activity on a 0–10 VAS and CRP level), SDAI score interpretation comprises also four categories: remission (0–3.3), low activity (3.4–11), moderate activity (11.1–26), and high RA activity (26.1–86).

Serological biomarkers (rheumatoid factor, RF, and ACPA) were evaluated only at baseline.

### 2.6. Periodontal Assessments

The periodontal status was recorded on a periodontal chart displaying the following clinical parameters for the entire dentition: number of present teeth, visible plaque index (VPI), gingival index (GI), bleeding on probing (BOP), probing pocket depth (PPD), and clinical attachment loss (CAL).

Clinical periodontal assessments were performed by a single trained examiner (C.I.) at the Sanocare Medical and Research Center, Iasi, who was blinded to the rheumatologic data. Access to previous assessment data was not allowed during the study. The examiner was considered calibrated when no statistically significant differences between measurements were obtained after the evaluation of 15 non-participant subjects on two occasions, one week apart. The mean values were assessed using paired *t*-test for VPI, GI, BOP, PPD, and CAL.

The periodontal evaluation was made in artificial light conditions, using a dental explorer, dental mirror, Williams probe, and air–water syringe.

VPI [58] or supragingival plaque was recorded dichotomously (present/absent) at 4 sites (mesial, distal, buccal, lingual/palatal) around each tooth. GI [59] assesses the gingival condition by gentle probing of the soft gingival wall at four sites for each tooth, as follows: 0 = absence of inflammation; 1 = mild inflammation: slight changes in color and texture, and slight edema, no bleeding on probing; 2 = moderate inflammation: redness, edema, glazing, bleeding on probing; 3 = severe inflammation: marked redness, edema, ulceration, tendency to spontaneous bleeding.

BOP evaluates gingival inflammation through bleeding observed 20 s after a probe is passed along inside the gingival sulcus or pocket. It was recorded dichotomously (present/absent) at 6 sites (mesiobuccal, mid-buccal, distobuccal, mesiolingual, mid-lingual, and distolingual) around each tooth.

PPD was measured between the gingival margin and the bottom of gingival sulcus or pocket, while CAL was measured between the cemento–enamel junction and the base of the gingival sulcus or pocket. Both parameters were determined with Williams periodontal probe and recorded on 6 sites per tooth. The recorded values were to the nearest millimeter, and every reading close to 0.5 mm was rounded to the nearest integer number.

PD was considered according to the case definition proposed by the 5th European Workshop on Periodontology in 2005 [60], as follows: level 1 (mild)—CAL ≥ 3 mm in 2 or more proximal sites of non-adjacent teeth and level 2 (severe)—CAL ≥ 5 mm in 30% or more proximal sites of teeth present; level 0 was considered—for healthy periodontal status or up to one proximal site with CAL ≥ 3 mm.

Patients were instructed to maintain their oral hygiene habits throughout the 6 months of follow-up; furthermore, as we intended to assess the accurate effect of TCZ on periodontal status, any periodontal treatment was avoided.

### 2.7. Statistical Analysis

Statistical analysis was performed with the IBM SPSS Statistics for Windows, Version 19.0. (IBM Corp., Armonk, NY, USA), with *p*-values less than 0.05 being considered as statistically significant; data at 3 and 6 months were summarized as means ± SD, or percentages (%) as appropriate, and correlations by Spearman rank tests; comparisons between baseline and 3 and 6 months of TCZ were assessed by Wilcoxon test.

## 3. Results

### 3.1. Baseline RA and Periodontal Assessments

Demographics, rheumatologic and periodontal characteristics, as well as RA-related drugs (concomitant glucocorticoids and immunosuppressives) taken at baseline are summarized in Table 1.

Most patients included in our study had seropositive established RA, with moderate-to-severe activity despite background medication. Eight patients (15.68%) received TCZ as their first biologic agent (bio-naïve), while the majority were bio-experienced patients, with failure (either insufficient response or adverse reactions) to previous biologics—15 (29.41%) to one biologic, 20 (39.21%) to two biologics, and 8 (15.68%) to three biologic agents.

We detected impaired oral health in all patients included in the final analysis, as follows: all had gingivitis (abnormal GI and increased prevalence of sites with BOP), and different degrees of chronic periodontitis (mainly level 1 and 2); advanced loss of attachment was reported in up to 23.52% of cases, while increased prevalence of sites with dental plaques in 21.56% of cases.

A closer look revealed a consistent positive correlation between the severity of chronic periodontitis, RA activity, and serum *r_2_* = 0.71 ACPA concentrations (*r_1_* = 0.81, *p_1_* = 0.001, *p_2_* = 0.002, respectively): the higher the RA activity and ACPA levels, the higher the PPD severity, with advanced CAL and tooth loss.

### 3.2. Changes in Rheumatologic and Periodontal Parameters with Tocilizumab

Changes in RA activity and periodontal status were reassessed after 3 and 6 months of TCZ; at follow-up visits, we reported significant improvement as compared to baseline (*p* < 0.05), although the results at 6 months were only slightly different from data obtained at 3 months (*p* > 0.05).

#### 3.2.1. Changes in Rheumatologic Status

Patients displayed consistent improvements in clinical activity meaning a significant decrease in the number of tender and swollen joints, VAS pain, and morning stiffness as rapid as 3 months; as expected, clinical response was maintained 3 months later in all patients, at the final monitoring visit. Similarly, we reported a dramatic decline in inflammatory biomarkers (both ESR and CRP), as well as a considerable immunologic response, particularly for serum levels of ACPA, but also for RF (Table 2).

DAS28-CRP and SDAI strongly improved during monitoring visits reaching either low disease activity or, even, remission (EULAR responders) vs. baseline, irrespective of the severity of periodontitis.

#### 3.2.2. Changes in Periodontal Status

Clinical data showed improvement in periodontal inflammation after only 3 months of TCZ and maintained over 6 months, as supported by an important decrease in gingival index and sites with bleeding of probing (*p* < 0.05). However, the improvement of specific periodontal parameters such as probing pocket depth becomes evident after prolonged treatment (6 months); overall, clinical attachment loss presented only slight changes without any statistical significance; teeth count and bacterial plaque scores were also not significantly influenced by medication (*p* > 0.05) (Table 3).

No significant correlations between changes in periodontal parameters and changes in RA activity were described in our study (*p* > 0.05).

We assumed that all the modifications in the degree of local gingival and periodontal inflammation is related to IL-6 blockade as no local periodontal treatment was allowed during follow-up.

## 4. Discussion

We aimed to assess the influence of the IL-6 receptor inhibitor on periodontal status in active RA associated with periodontitis, assuming that TCZ might be able not only to improve clinical and biochemical RA-related parameters but also to ameliorate chronic periodontitis as a result of decreased IL-6 in the periodontal microenvironment via declining systemic inflammation.

Although our target is to demonstrate the ability of TCZ to modulate periodontal inflammation and subsequent damage, firstly, we emphasized its role in controlling RA activity. We reasonably confirmed a consistent response to TCZ in real-life settings, which was achieved in as rapid as three months and continued after six months of therapy, irrespective of background medication and clinical scenario (mono- or combined therapy, bio-naïve or bio-experienced patients); it is more than clear that even in the short-term, IL-6 blockade displays significant clinical, biological, as well as serologic disease improvement. Although we found no consistent difference in clinical response in seropositive vs. seronegative RA, we noticed a significant impact on ACPA serum concentration after six months, which is an improvement that parallels the decrease in periodontal inflammation, suggesting a role of IL-6 in both systemic and local inflammation (synovial and periodontal) and the potential implications via citrullination. Therefore, our results stand by as a proof of the effectiveness of subcutaneous TCZ in managing inflammatory and immune pathways in RA [53].

We also focused on the magnitude of compromised oral health in RA; most patients in our initial group presented a high rate of mild and severe periodontal disease, validating/reinforcing the already known risk of periodontitis in such patients, particularly in established, longstanding disease [2,3,4,9,37]. We have included in the final analysis only those cases with overt periodontal disease, meaning that up to 75% had at baseline altered periodontal status in a group of consecutive patients starting TCZ for their active disease. Indeed, recent reviews and meta-analyses have already discussed periodontal disease in various RA settings (independent of age, disease duration, serology profile, and disease activity) compared to general population [1,3,5,11,19,20,21,23].

We identified excessive gingival involvement confirmed by an increased percentage of sites with plaques and inflammation and abnormal periodontal status (e.g., increased probing depth, clinical attachment loss) supporting data from the literature [6,16,17,20,21,27,28,38,39,44,48]. Moreover, we recognized positive correlations between the severity of periodontitis, inflammatory parameters (especially CRP), serology (ACPA status and titers), and RA activity; indeed, recent studies suggest a worse periodontal status in active untreated RA, and higher CRP if RA is associated with severe periodontitis [6,11,15,16,17,19,27,28,37]. Finally, it seems that ACPA-positive patients had severely impaired periodontal health, while disease activity correlated with periodontitis degree as well [6,16,17,19,27,28,37].

Finally, we demonstrated that short-term tocilizumab significantly reduced gingival as well as periodontal inflammation as supported by decreased levels of gingival index, bleeding on probing, and probing pocket depth, paralleling the articular improvement. Indeed, only minor changes in clinical attachment loss were detected in our enrolled patients, and the supragingival plaque remained stable after 3 and 6 months of biological treatment (*p* > 0.05).

A closer look at recent data definitely emphasizes the dual effects of early and aggressive RA treatment with biologic and non-biologic drugs (Janus kinase inhibitors, JAK inhibitors) on articular as well as comorbid periodontal disease [5,8,12,16,17,25,27,28,37,38,39]. It is widely accepted that TNF and IL-6 receptor inhibitors are able to ameliorate oral health in active RA, as reflected by clinical, biological, and even serological RA biomarkers [5,16,17,27,28,38,39,44,45,46]. Although there are controversial effectiveness signals with TNF inhibitors in improving chronic periodontitis [3,6,11,15,19,23,24,25,26,33,38,39], all papers about anti-IL-6 therapy clearly demonstrated articular, systemic, and also periodontal benefits with TCZ without any periodontal specific treatment [16,17,27,28].

An interesting trial compared periodontal condition in patients with RA and periodontitis before and after biological therapy in two cohorts: one under tocilizumab and the other receiving medication with TNF inhibitors [16]. After 6 months, both tocilizumab and TNF inhibitors demonstrated a consistent improvement of oral health with significantly reduced periodontal inflammation (gingival index, bleeding on probing, and probing depth) compared to baseline, with similar results in both cohorts unless there was a greater decrease in gingival index and less gingival inflammation with tocilizumab; however, plaque levels remained the same irrespective of medication, while periodontal clinical attachment loss decreased only after TCZ but not after TNF inhibitors [16,17,27,28]. These observations were partially supported by the results of another study about an excessive inflammatory response against oral pathogens essentially based on high levels of IL-6 [16,17,27,28,31].

Recent meta-analyses reviewed the most important studies on TNF and non-TNF biologics in patients with RA and PD [5,20]. The critical difference between the class of TNF inhibitors and TCZ or B-cell depletive agent rituximab is that infliximab, an anti-TNF monoclonal antibody, may negatively address gingival inflammation although it may also improve alveolar bone destruction [5,6,7,19,37] resulting in a dissociated response for patients with severe periodontitis [5], while both tocilizumab and rituximab associate with significant a down regulation of gingival inflammation and damage in RA associated with periodontitis [16,17,27,28,50].

Additional research is necessary to clearly differentiate between the direct effects of TCZ on local periodontal inflammation and IL-6 or its receptor levels in the gingival crevicular fluids and periodontium of patients and the indirect effect via dramatically decreasing systemic inflammation, which may impact also oral health [16,17,27,28]. Indeed, numerous studies indicated a rapid and significant decline in typical inflammatory parameters (ESR and CRP), but also in serological RA biomarkers (RF, ACPA) as well as inflammatory cytokines (TNF, IL-6) and mediators (serum-amyloid A, matrixmetaloproteinases 1, 3), supporting the indirect role of TCZ in periodontitis [16,17,27,28].

In our study, we assessed specific gingival and periodontal parameters before and after short-term TCZ therapy. We demonstrated successful RA as well as periodontal outcomes with TCZ and independent of potential confounding factors (such as smoking, diabetes, hematological conditions, sex steroid hormones elevations, pharmacological agents) related to periodontal disease, as such patients were excluded from the final analysis.

We concluded that tocilizumab decreased gingival inflammation since no periodontal therapy was permitted and the dental hygiene behavior remained unchanged in our enrolled patients.

Unfortunately, we were not able to assess either the serum or gingival crevicular fluid levels of IL-6 or its receptor in all patients; we assumed that tocilizumab indirectly contributes to modulate local (gingival and periodontal) inflammation by limiting systemic inflammation. Indeed, the biofilm plaque accumulation was not consistently diminished with tocilizumab, and we were not able to depict a spectacular impact on clinical attachment loss, but we arrived to demonstrate a positive effect of IL-6 blockade on exuberant gingival inflammation.

Further studies are necessary to confirm the benefits of Il-6 inhibitors in larger populations and longer follow-ups also focusing on IL-6/IL-6 receptor levels in gingival crevicular fluid.

## Figures and Tables

**Table 1:** Demographic, rheumatologic, and periodontal characteristics at baseline.

Baseline Parameters	Baseline
**Demographics**	
Age (years; mean ± SD)	56.3 ± 15.7
Female (*n,* %)	46 (90.1)
**RA-related parameters**	
Duration of RA (months; mean ± SD)	81.3 ± 68.9
DAS28-CRP (mean ± SD)	5.36 ± 1.67
SDAI (mean ± SD)	34.2 ± 16.3
Corticosteroids (*n*, %)	20 (39.21)
DMARDs (*n,* %)	46 (90.1)
ACPA levels (U/mL; mean ± SD)	239.7 ± 124.3
ACPA positivity (*n,* %)	32 (62.74)
RF levels (IU/mL; mean ± SD)	192.7 ± 85.3
RF positivity (*n,* %)	47 (92.15)
Serum CRP levels (mg/dL; mean ± SD)	15.3 ± 6.9
**PD-related parameters**	
Number of present teeth (mean ± SD)	23.7 ± 3.4
GI (mean ± SD)	0.98 ± 0.12
% sites with plaque (mean ± SD)	32.4 ± 16.9
% sites with BOP (mean ± SD)	10.2 ± 8.6
PPD (mm; mean ± SD)	2.8 ± 0.4
% sites with PPD ≥ 4 mm	12.7 ± 2.5
CAL (mm)	3.5 ± 1.2
% CAL ≥ 3 mm	12.5 ± 0.2

RA, rheumatoid arthritis; DAS28-CRP, Disease Activity Score on 28 joints using C-reactive protein; SDAI, Simplified Disease Activity Index; DMARDs, disease-modifying antirheumatic drugs; ACPA, anti-citrullinated protein antibodies; RF, rheumatoid factor; PD, periodontal disease; GI, gingival index; BOP, bleeding on probing; PPD, probing pocket depth; CAL, clinical attachment loss; n, number; SD, standard deviation; %, percent.

**Table 2:** Changes in rheumatoid arthritis (RA)-related parameters at 3 and 6 months after tocilizumab.

Parameter	Baseline	3 Months (V1)	6 Months (V2)	*p*-Value
DAS28-CRP (mean ± SD)	5.36 ± 1.67	3.39 ± 0.57	2.41 ± 0.19	* <0.05; ** <0.05
SDAI (mean ± SD)	34.2 ± 16.3	18.1 ± 8.2	11.1 ± 4.3	* <0.05; ** <0.05
Number of tender joints (mean ± SD)	12.31 ± 4.29	4.56 ± 1.31	3.55 ± 1.13	* <0.05; ** NS
Number of swollen joints (mean ± SD)	10.01 ± 3.37	2.85 ± 4.22	1.50 ± 2.09	* <0.05; ** NS
Pain VAS mm (mean ± SD)	82.7 ± 21.5	28.8 ± 23.2	16.3 ± 11.8	* <0.05; ** <0.05
Serum anti-CCP titer (U/mL) (mean ± SD)	239.7 ± 124.3	192.6 ± 112.4	123.6 ± 101.6	* <0.05; ** NS
Serum RF levels (IU/mL) (mean ± SD)	192.7 ± 85.3	164.8 ± 92.5	151.7 ± 89.3	* NS; ** NS
Serum CRP levels (mg/dL) (mean ± SD)	15.3 ± 6.9	4.12 ± 0.92	3.92 ± 0.34	* <0.05; ** NS

SD; standard deviation; DAS28-CRP, Disease activity score on 28 joints based on C-reactive protein; SDAI, Simplified Disease Activity Index; VAS, 0–10 cm visual analogue scale; CCP, cyclic citrullinated peptide; RF, rheumatoid factor; V, visits; * V1 compared to baseline; ** V2 compared to V1; NS, non-significant (0.05).

**Table 3:** Changes in PD-related parameters at 3 and 6 months after tocilizumab.

Parameter	Baseline	3 Months (V1)	6 Months (V2)	*p*-Value
GI	0.98 ± 0.12	0.85 ± 0.17	0.81 ± 0.18	* <0.05; ** NS
% sites with plaque	32.4 ± 16.9	30.5 ± 14.2	30.2 ± 15.8	* NS; ** NS
% sites with BOP	10.2 ± 8.6	7.3 ± 6.1	6.5 ± 6.8	* <0.05; ** NS
PPD (mm)	2.8 ± 0.4	2.1 ± 0.12	2.1 ± 0.09	* <0.05; ** NS
% sites with PPD ≥ 4 mm	12.7 ± 2.5	7.8 ± 3.9	6.1 ± 3.6	* <0.05; ** NS
CAL (mm)	3.5 ± 1.2	2.58 ± 0.30	2.55 ± 0.31	* <0.05; ** NS
% sites with CAL ≥ 4 mm	12.5 ± 0.2	11.2 ± 0.4	11.3 ± 0.9	* <0.05; ** NS

GI, gingival index; BOP, bleeding on probing; PPD, probing pocket depth; CAL, clinical attachment loss; * V1 compared to baseline; ** V2 compared to V1; *p* > 0.05 non-significant (NS); V1 and V2, visit 1 and 2, respectively.
